# Evaluation of Darkroom disease’s symptoms among radiographers in the West Bank hospitals: a cross-sectional study in Palestine

**DOI:** 10.1186/1745-6673-9-15

**Published:** 2014-04-17

**Authors:** Hamzeh Al Zabadi, Yaser Nazzal

**Affiliations:** 1 Public Health Department, Faculty of Medicine and Health Sciences, An-Najah National University, PO.Box 7, West Bank, Nablus, Palestine; 2 Master of Public Health Program, Faculty of Graduate studies, An-Najah National University, Nablus, Palestine

## Abstract

**Background:**

Radiographers report many unexplained work related symptoms attributed to “darkroom disease symptoms” such as headache, skin rash, mouth sores, blurred vision, palpitation, and chemical taste. The aim of the present study was to assess the prevalence of occupationally-related darkroom disease symptoms among male radiographers in the West Bank hospitals.

**Methods:**

A cross sectional study was conducted on a non-random purposive sample of male radiographers (study group) and nurses (control group) using a previously validated and standardized questionnaire.

**Results:**

We were able to recruit 330 radiographers and 242 nurses. Data analysis showed that the majority of both groups aged between (36–43) years. Furthermore, the differences in the reported prevalence of symptoms among radiographers showed a statistically significant higher percentage for each reported symptom compared to nurses (P-values <0.001). In multivariate linear regression, staying more than 30 minutes in the darkroom per shift was associated with a significant increase in the mean number of reported symptoms (P-value < 0.001). However, the availability of a ventilating machine in the darkroom showed a strong negative association with the mean number of reported symptoms (P-value < 0.001).

**Conclusions:**

Our findings could help overcome the limitations usually encountered in such complex occupational exposure. However, trying to interpret our finding directly to chemicals exposure in the radiographers’ occupational setting should be done with caution due to the absence of active or passive monitoring for the suspected chemicals.

## Background

In many occupational settings, some chemicals could adversely affect health and contaminate the environment [1]. Extensive use of x-ray processing chemistry on a world**-**wide basis has raised professional concerns regarding darkroom disease which is a term used to describe unexpected multiple symptoms attributed by radiographer to their work environment when being exposed to film processing chemicals [2-6]. These symptoms include: headaches, skin rashes, shortness of breath, mouth ulcers, unusual heart rhythms, painful joints, runny/stuffy nose and nausea.

X-rays could create a latent image on the film surface by reducing the silver halide crystals to elemental silver then the image is amplified and stabilized during the development process using agents such as hydroquinone. The image is fixed by agents, which dissolve and remove the unused silver halides [7]. Automated x-ray film processing machines achieve short development times by using elevated temperatures (28-35°C), by including glutaraldehyde as a hardening agent within the developer solution, and by actively drying the fixed and washed film with heated air [8]. This process of radiographic film development might therefore induce potential exposures to hydroquinone, glutaraldehyde, formaldehyde, glycols (diethylene, triethylene), acetic acid, sodium sulphite, sulfur dioxide, ammonium chloride, silver compounds, 5-nitroindazole, Thiosulphate, 1-phenyl-3-pyramzolidone and other chemicals [9-12].

Radiography departments could have poor structural designs together with operational deficiencies of ventilation system and closed/dark workplace [13]. During manual film processing, cleaning of the internal components of the film processor or during the normal processing procedures, radiographers might be exposed to the above mentioned chemicals through either direct or indirect skin contact, fumes inhalation or via ingestion. Therefore, this exposure in such an occupational setting is complex and implies multiple chemicals. Consequently, it is not appropriate to assess the exposure to a single chemical as the outcomes could be related to the overall synergistic and pharmacokinetics interactions between these chemicals in the human body. Worldwide, few studies have been conducted on the radiographers in order to clarify the link between their exposures and the workplace related symptoms [14]. As an expected outcome, this study will assess the prevalence of occupationally-related darkroom disease symptoms among the radiographers (exposed group) compared to the nurses (non-exposed group) in the West Bank hospitals in order to implement preventive and effective protocols for the control of this occupationally-related disease. The results of this study will improve our understanding in a way that might help overcome the limitations of environmental exposure assessment in such a very complex occupational setting.

## Methods

### Study design and population

A cross sectional study was conducted. The study population involved subjects recruited from the two professional health team members; the radiographers (exposed group) and the hospital’s nurses (non-exposed group) selected from the chosen Palestinian governmental and non-governmental hospitals listed in Table 1. As most of the radiographers personnel were males, only male nurses were included in the study to avoid bias from gender differences. Both study populations were selected from the same occupational setting, from similar demographic category, had worked in the field since at least one year and agreed to participate. However, those with physician diagnosed asthma before their current occupation were excluded, as asthma symptoms could interfere with the study outcomes (i.e. darkroom disease symptoms). Although random sampling would have been more appropriate, our method of recruitment could have limited our selection and classification biases to the minimum as well.

**Table 1:** The study selected hospitals stratified by governorate, and sector (governmental and non-governmental)

**Governorate**	**Governmental hospitals**	**Non-governmental hospitals**
Ramallah	Al-Mujamaa’ Al-Tibi	Red Crescent society; Arab Medical Care; Al-Mustaqbal
Jenin	Martyer Dr. Khalil Suleiman	Al-Razi; Patient Friend Society
Tulkarm	Thabit Thabit	Al-Zakat; Red Crescent Society
Nablus	Rafedia; Al-Watani	Al-Injili; Al-Ittihad; Al-Arabi Al-Takhasussi
Qalqilya	Darwish Nazzal	Al-Aqsa
Salfit	Salfit	*None
Jericho	Jericho	*None
Bethlehm	Alhussein	*None
Hebron	A'alia	Alahli

*None: No non-governmental hospitals in these Governorates.

### Study settings

The study was conducted in the x-ray departments in the governmental and non-governmental hospitals in the West Bank over 3 months period (1^st^ January-1^st^ April 2012). The selected hospitals were chosen primarily because they have large numbers of radiographers and therefore are representative of the most Palestinian hospitals in the West Bank.

### Sample size

There were nearly (518) radiographers who currently (at the time of study) work in the x-ray departments all over the Palestinian hospitals in the West bank. Out of them, 471 are males. About 330 radiographers were selected of all those met the selection criteria and agreed to participate using non-random purposive sampling technique. Similarly, the male nurses (n = 242) responded by the same sampling method from the same hospitals assuring that the selection criteria are fit. Based on the study type І error (α) that has been estimated up to 5% for the study and a power expectation of 80%, a sample size of 250 in each group was found to be sufficiently large enough to highlight the expected differences (10%) between the two study groups.

### Ethical considerations

The study protocol was approved by the Institutional Review Board (IRB) and the scientific research committee of the Public Health Department as well as the Faculty of Graduate Studies Scientific Research Board at An-Najah National University. Permissions to conduct the study in the hospitals’ x-ray departments were obtained from the Palestinian Ministry of Health (MoH) for the governmental hospitals and from the hospital’s managers for the non-governmental hospitals. A written and signed informed consent was obtained from each participant who met the selection criteria and agreed to participate voluntary.

### Data collection

Data were collected by using a standardized and a previously-validated face-to-face administered questionnaire adapted with permission from Damases, (2006) [15]. In each hospital, male radiographers available at that time who met the inclusion criteria and where able to sign the consent form voluntary were interviewed. The interview was in private with each radiographer and in a face-to-face assuring that every question in the questionnaire was answered and clear. After conducting x-ray departmental questionnaires, face-to-face interviews with the available male nurses in the nursing wards at that time were conducted in the same manner taking into consideration equal numbers with radiographers (although this was not always the case).

The questionnaire included questions dealing with the study independent and dependant variables. It included; (1) Socio-demographic factors and exposure to some factors that might influence health such as smoking status and habits, (2) Exposure to external factors such as living near to industrial areas and sharing home with people who smoke, (3) A self-reported list of symptoms of darkroom disease such as; headache, nausea, irritation of throat, sneezing or nose itchy (not including common cold), heart beating abnormally and others, (4) Questions for radiology workers only covered the availability and conditions of services such as; performing daily radiographic images and duration time spent in the darkroom per working shift; availability of windows and more than one door, availability of ventilating machine and exhaust to transmit the fumes outside the darkroom.

### Pilot testing of the questionnaire

The questionnaire was piloted before using in the field and on the Palestinian population. Indeed, ten radiographers and ten nurses (who work in the male wards in Jenin hospitals) were asked to fill in the questionnaires in order to examine its clarity and comprehensiveness for the Palestinian population. As a result of this pre-test (pilot testing), no major changes were found to be necessary. However, minor modifications were judged necessary to improve the questionnaire clarity and presentation of questions for the local Palestinian conditions. The data gathered from the pilot testing was not included in the main study.

### Data analysis

All data were analyzed using the statistical software 16 package SPSS (Statistical Package for the Social Sciences) [16]. Chi-square test for trends was carried out to analyze the differences between the dependant variable and the qualitative independent variables and in regard to the percentages of the reported symptoms. Students**-**t test and ANOVA were used to test the mean differences of the continuous dependant variable (number of symptoms) among different categorical independent variables. Multivariate linear regression analysis adjusted for possible confounders was developed to assess the associated occupational factors with the mean number of symptoms among radiographers (out of 20 studied symptoms). *P*-value less than 0.05 was always considered significant. In the questionnaire, there were 20 questions about symptoms, and the answers’ categories for each question were (yes, no). These questions were used to develop new variables with scorings. Each score was the sum answer “yes” for each of the 20 symptoms’ questions. Therefore, the score ranged from 0–20. This new variable was then analyzed as continuous variable in the analysis.

We have conducted a one-Way ANOVA analysis for all questionnaire variables (those with two and those with more than two categories. However, variables that where more than two categories and showed significant associations in this stage of analysis with the mean number of symptoms (out of 20 total symptoms) were categorized again into two categories and re-tested for significance using the same ANOVA analysis. Indeed, all that were significant on more than two categories remained also significant after re-categorization into two categories (data available upon request).This process of re-categorization of some variables where done due to the large number of variables that showed a significant association with the mean number of symptoms in the bivariate analysis. Therefore, and for the purpose of developing not an overloaded multivariate linear regression model, we have categorized the variables with more than two categories into two categories. Then, we have calculated the mean number of symptoms (out of 20 total symptoms) among each variable category in the radiographers’ subjects in order to predict the variables that could be associated with the mean number of symptoms among those subjects (radiographers).

Variables entered in the final multivariate regression model were those with a significant P value of less than or equal 0.05. However, the variables “number of working hours-less than or equal 8 hrs/more than 8 hrs; is there a window in the darkroom-no/yes; and is there an exhaust in the darkroom-no/yes” were also entered in the final model although they were not significant in the first stage of analysis due to their possible effects and associations with the model dependant variable “number of symptoms among radiographers” after adjusting for other variables.

## Results

### Characteristics of the study population

In this study, we were able to recruit 572 participants from both groups (radiographers and male nurses). The radiographers were 330 subjects who represent nearly 57.7% of all population. However, the male nurses were 242 participants representing nearly 42.3% of all population in both groups. The distribution of the study population is shown in Table 2 below.

**Table 2:** Description of the study subjects

**Variable**	**Total**	**Current occupation**		***P*-Value**
		n (%)*		
	N (%)* = 572	**Radiographers**	**Nurses**	
		n (%)*	n (%)*	
Age				
20-27years	106 (18.5)	60 (18.2)	46 (19)	0.092
28-35 years	154 (26.9)	88 (26.7)	66 (27.3)	
36-43 years	160 (28)	92 (27.9)	68 (28.1)	
44-51 years	124 (21.7)	80 (24.2)	44 (18.2)	
>51 years	28 (4.9)	10 (3)	18 (7.4)	
Marital status				
Single	152 (26.6)	86 (26.1)	66 (27.3)	0.942
Married	410 (71.7)	238 (72.1)	172 (71.1)	
Widower	10 (1.7)	6 (1.8)	4 (1.6)	
Educational level				
Diploma	236 (41.3)	104 (31.5)	132 (54.5)	0.000**
Bachelor	316 (55.2)	216 (65.5)	100 (41.3)	
Master	14 (2.4)	8 (2.4)	6 (2.5)	
PhD	6 (1)	2 (0.6)	4 (1.7)	
Monthly net income				
1500-2000NIS	84 (14.7)	36 (10.9)	48 (19.8)	0.005**
2001-2500NIS	382 (66.8)	236 (71.5)	146 (60.3)	
2501-3000NIS	106 (18.5)	58 (17.6)	48 (19.8)	
Residence place				
City	210 (36.7)	124 (37.6)	86 (35.5)	0.018**
Village	268 (46.9)	164 (49.7)	104 (43)	
Refugee camp	94 (16.4)	42 (12.7)	52 (21.5)	
Type of hospital				
Non-Governmental	302 (52.8)	160 (48.5)	142 (58.7)	0.016**
Governmental	270 (47.2)	170 (51.5)	100 (41.3)	
Duration in current occupation				
1-5years	218 (38.1)	126 (38.2)	92 (38)	0.001**
6-10years	126 (22)	64 (19.4)	62 (25.6)	
11-15years	128 (22.4)	74 (22.4)	54 (22.3)	
>15 years	100 (17.5)	66 (20)	34 (14)	
Daily working hours				
<8hours	120 (21)	88 (26.7)	32 (13.2)	0.001**
8hours	394 (68.9)	222 (67.3)	172 (71.1)	
> 8hours	58 (10.1)	20 (6)	38 (15.7)	
Do you live in an industrial area?				
Yes	84 (14.7)	66 (20)	18 (7.4)	0.001**
No	488 (85.3)	264 (80)	224 (92.6)	
Do you share your home with people who smoke?				
Yes	118 (20.6)	72 (21.8)	46 (19)	0.412
No	454 (79.3)	258 (78.2)	196 (81)	
Smoking status				
Current smoker	166 (29)	86 (26.1)	80 (33.1)	0.176
Ex-smoker	38 (6.6)	24 (7.3)	14 (5.8)	
Non-smoker	368 (64.4)	220 (66.7)	148 (61.2)	

*Data are expressed as number (percent) of each group. **Statistically significant (p <0.05).

As shown in the Table 2, the largest number of respondents among radiographers and nurses indicated their age as between “36-43” years (n = 92 and 27.9%; n = 68; 28.1%; respectively). No statistically significant differences were found between the study groups regarding the age and the marital status. However, a significant relationship was found between the study groups in regard to the educational level where nearly 65.5% and only 41.3% of radiographers and nurses (respectively) reported having a Bachelor degree. The other remaining factors showed statistically significant differences between the two study groups (for more details, see Table 2).

### Exposure to internal and external factors

As shown in the Table 2 below, participants were also asked to offer information concerning their neighborhood residence. Nearly only 20% of radiographers (n = 66) reported that they reside in an industrial area, while about only 7.4% of nurses (n = 18) reported that they live in such areas with a statistically significant relationship between current occupation and neighborhood locality. On the other hand, there were no statistically significant relationship between current occupation and sharing home with people who smoke neither there was in regard to smoking status (for more details, see Table 2).

### Evaluation of darkroom disease’s symptoms

We have evaluated the darkroom disease’s symptoms as the main study objective. Table 3 below shows the self-reported frequency (percent) of darkroom disease’s symptoms among the two study groups (radiographers and nurses). As shown in the Table 3, the differences in the reported percentage of symptoms among radiographers showed a statistically significant higher percentage for each reported symptom compared to the nurses (P-values <0.001; see Table 3 for the remaining symptoms).

**Table 3:** The self-reported frequency (percent) of darkroom disease’s symptoms among radiographers and nurses in the past six months

**Symptom**	**Radiographers**	**Nurses**	**Chi-square**
	n (%)*	n (%)*	***P*-value**
Headache	250 (75.8)	142 (58.7)	< 0.001**
Nausea	170 (51.5)	36 (14.9)	< 0.001**
Runny nose	208 (63)	38 (15.7)	< 0.001**
Irritation of throat	228 (69.1)	50 (20.7)	< 0.001**
Unexpected fatigue	216 (65.5)	88 (36.4)	< 0.001**
Ringing in the ears	184 (55.8)	38 (15.7)	< 0.001**
Lip sores	136 (41.2)	42 (17.4)	< 0.001**
Mouth sores	142 (43)	26 (10.7)	< 0.001**
Heart beating abnormally	116 (35.2)	38 (15.7)	< 0.001**
Unusual numb arms and legs	162 (49.1)	60 (24.8)	< 0.001**
Skin rash	196 (59.4)	26 (10.7)	< 0.001**
Abdominal pain	160 (48.5)	64 (26.4)	< 0.001**
Blurred vision	136 (41.2)	42 (17.4)	< 0.001**
Dizziness	154 (46.7)	52 (21.5)	< 0.001**
Runny eyes	146 (44.2)	34 (14)	< 0.001**
Night sweat	98 (29.7)	18 (7.4)	< 0.001**
Palpitation	102 (30.9)	28 (11.6)	< 0.001**
Urination pain	106 (32.1)	34 (14)	< 0.001**
Chemical taste	202 (61.2)	0 (0)	< 0.001**
Sneezing/nose itchy	234 (70.9)	40 (16.5)	< 0.001**

*Data are expressed as number (percent) of each positive answer (yes) to each symptom. The non presented data equals the negative answer (No). **Statistically significant (p <0.05).

The specific health symptoms of the radiographers and nurses are mentioned in Table 3. The most predominant health symptoms in descending order of frequency, addressed by radiographers were: headache (75.8%), sneezing/nose itchy (70.9%), irritation of throat (69.1%), unexpected fatigue (65.5%), runny nose (63%), chemical taste (61.2%), skin rash (59.4%), ringing in the ears (55.8%), nausea (51.5%), unusual numb arms and legs (49.1%), abdominal pain (48.5%), dizziness (46.7%), runny eyes (44.2%), mouth sores (43%), lip sores (41.2%), blurred vision (41.2%), heart beating abnormally (35.2%), urination pain (32.1%), palpitation (30.9%) and finally night sweat (29.7%).

### Evaluation of occupational conditions for radiographers

Table 4 below shows some of the occupational conditions for the radiographers. The frequency of radiographers and the number of symptoms among each different category of an occupational condition are shown below. As shown in the table, the majority (63%) of the radiographers reported performing more than 15 images per day and the higher number of performed images per day the higher number of symptoms reported as well. On the other hand, nearly 104 (31.5%) gave an account of spending (1–30) minutes in the darkroom during the working shift, while the minority (16.4%) stated of spending more than 90 minutes per working shift. The vast majority of radiographers (n = 292; 88.5%) reported not having windows in the darkrooms. Moreover, most of the radiographers (n = 254; 77%) indicated not having alternative/additional door in the darkroom and about 47.3% of them reported not having ventilating machines in the darkrooms, whilst 74.5% notified not having an exhaust to transmit the fumes and odors outside the darkroom. Clearly and as shown in Table 4, the radiographers were more likely to report symptom clusters associated with working factors expected to reflect greater workplace chemical exposures and symptoms; less local exhaust of machines, less frequent adequate ventilation in the processing area, intense load of images done daily, elongated time spent in the darkroom, low accessibility of a window and an extra door in the darkroom.

**Table 4:** Occupational condition variables by number (percent) of subjects and number (percent) of total reported symptoms (3346) among radiographers (n = 330)

**Working condition variables**	**Radiographers**	**Number of symptoms among radiographers**
	n (%)*	**No. (%)**^**!**^
How many radiographic images do you perform every day?		
1-5 images	28 (8.5)	290 (8.7)
6-10 images	10 (3)	30 (0.9)
11-15 images	84 (25.5)	756 (22.6)
>15 images	208 (63)	2270 (67.8)
The time spent in the darkroom during the working shift?		
1-30 minutes	104 (31.5)	1002 (29.9)
31-60 minutes	72 (21.8)	660 (19.7)
61-90 minutes	100 (30.3)	1048 (31.3)
> 90 minutes	54 (16.4)	636 (19)
Is there a window in the darkroom?		
Yes	38 (11.5)	444 (13.3)
No	292 (88.5)	2902 (86.7)
Does the darkroom have more than one door where you work?		
Yes	76 (23)	444 (13.3)
No	254 (77)	2902 (86.7)
Is there a ventilating machine in the dark room where you work?		
Yes	174 (52.7)	1566 (46.8)
No	156 (47.3)	1780 (53.2)
Is there an exhaust to transmit the fumes outside the darkroom?		
Yes	84 (25.5)	898 (26.8)
No	246 (74.5)	2448 (73.2)

*Data is expressed as number (percent) for each variable’s category. ^!^Number of symptoms (percent from the total number of symptoms reported among radiographers; 3346).

### Multivariate analysis of the mean number of symptoms

The final multivariate linear regression model with all possible predictors is shown in the Table 5. As shown in Table 5, the monthly income was a significant predictor for the mean number of symptoms with a positive association. However, living in a village, reporting living in an industrial area (yes), reporting sharing home with people who smoke (yes), the years of experience (more than 10 years) showed a significantly positive association with the mean number of reported symptoms.

**Table 5:** Multivariate linear regression model* for the association of the mean number of symptoms with some possible predictors among radiographers (N = 330)

**Independent variables**	**B**	**SE**	**Beta**	***P-*** **value**	**95% CI for B**
Age (20–40 years/>40 years)	0.92	0.66	0.07	0.16	(−0.37-2.22)
Monthly net income (≤2500/>2500) NIS	2.35	0.70	0.14	0.001	(0.96-3.74)*
Residence place (city and refugee camp/village)	1.15	0.52	0.09	0.03	(0.12-2.19)*
Years of experience (1–10 years/>10 years)	1.31	0.59	0.10	0.03	(0.15-2.47)*
Daily working hours (≤8 hours/>8 hours)	0.51	1.11	0.02	0.65	(−1.68-2.69)
Living in industrial area (no/yes)	5.63	1.13	0.37	0.001	(3.39-7.86)*
Sharing home with people who smoke (no/yes)	3.79	1.12	0.25	0.001	(1.57-6.004)*
Daily images performed per working shift (≤15 images/>15 images)	−0.12	0.65	−0.009	0.85	(−1.39-1.16)
Period of stay in darkroom per shift (1–30 minutes />30 minutes)	3.28	0.62	0.27	0.001	(2.06-4.51)*
Availability of a window in the darkroom (no/yes)	1.58	1.11	0.08	0.15	(−0.61-3.77)
Availability of an extra door in the darkroom (no/yes)	1.19	0.80	0.08	0.13	(−0.38-2.77)
Availability of a ventilating machine in the darkroom (no/yes)	−1.98	0.54	−0.16	0.001	(−3.05- -0.91)*
Availability of an exhaust in the darkroom (no/yes)	−0.57	0.64	−0.04	0.37	(−1.83-0.68)

*Variables entered in the model are those with a P-value of <0.05 in One-way ANOVA. Number of worked hours per day, availability of exhaust, availability of window in the darkroom were entered in the model although not significant in the bivariate analysis; NIS, New Israeli Shekels; SE, standard error; B, unstandardized regression coefficient; Beta, standardized regression coefficient; CI, confidence interval. *Statistically significant (p <0.05). Enter regression method was used. R for the model = 0.71; Adjusted R square = 0.46 (R^2^ = 0.477; P value of the overall significance of regression model <0.001).

Regarding some occupational factors, the period of stay in the darkroom per shift showed a strong significant association with the mean number of reported symptoms (i.e., reporting staying more than 30 minutes in the darkroom per shift was associated with a significant increase in the mean number of reported symptoms). However, the availability of a ventilating machine in the darkroom showed a strong negative association with the mean number of reported symptoms (i.e., reporting having a ventilating machine in the darkroom was associated with a significant decrease in the mean number of reported symptoms). All other variables did not remain significant after adjusting for other variables in the model.

## Discussion

The aim of the present study was to assess the prevalence of occupationally-related darkroom disease symptoms among the radiographers (exposed group) compared to the nurses (non-exposed group) in the Palestinian hospitals in the West Bank. The main study findings was that, the reported prevalence of symptoms among radiographers showed a statistically significant higher percentage for each reported symptom compared to the nurses (P-values <0.001). In multivariate linear regression analysis, the monthly income was a significant predictor for the mean number of symptoms with a positive association among radiographers. However, living in a village, reporting living in an industrial area (yes), reporting sharing home with people who smoke (yes), the years of experience (more than 10 years) showed a significantly positive association with the mean number of reported symptoms among radiographers.

Regarding some occupational factors, the period of stay in the darkroom per shift showed a strong significant association with the mean number of reported symptoms among radiographers (i.e., reporting staying more than 30 minutes in the darkroom per shift was associated with a significant increase in the mean number of reported symptoms). However, the availability of a ventilating machine in the darkroom showed a strong negative association with the mean number of reported symptoms among radiographers.

### Participants’ socio-demographic factors

The possible reasons which could have contributed to a lower response rate for both groups are scheduling of examinations, vacation leave, sick leave, resignation and scheduling of work shifts. Nurses were chosen as the non-exposed group. There were some significant differences between the exposed and non-exposed group in some aspects like monthly income, educational level, duration in the current occupation and daily working hours (see Table 2 for more details). Due to large number of outcome variables (symptoms) compared between radiographers and nurses (Table 3) and not to overload the data with many adjusted models, we only described the univariate differences in the percentages of symptoms between the two groups. However, when we developed our final multivariate model of the symptoms, we have adjusted for all these possible confounders (see Table 5 for details). Furthermore, no significant differences in age and marital status were noted between the two groups and both groups showed a statistical similarity regarding sharing home with people who smoke and smoking status variables.

### Evaluation of darkroom disease’s symptoms among radiographers and nurses

In Palestine, data about darkroom disease symptoms among radiographers are lacking. The present study has found that radiographers have a total of 3346 symptoms, while nurses count for about 896 symptoms in cumulative. These symptom clusters were significantly more common among radiographers than among nurses, occurring over four times as often and are consistent with previous studies of darkroom disease symptoms [4,10]. The most significant symptoms measured in the exposed group were headache, sneezing/nose itchy, irritation of throat, unexpected fatigue, runny nose and chemical taste. In addition to these common symptoms, the exposed group also reported chest illness, nausea, painful joints, ringing ears, skin rash, lip sores, mouth sores, abnormal heart beat and numbness of arms and legs.

It is suggested that these darkroom disease symptoms clusters could be related to exposure to high air concentrations of chemicals (this should be mentioned with caution in this study as we didn’t perform an air sampling of the workplace). However, the findings of this study are in accordance with those found in other studies [6] on medical radiation technologist which found that sore throat, headache, sore or itchy eyes, abnormal heart beating and runny nose were significant symptoms compared to non-exposed group. The respiratory problems reported in this study are in parallel with other studies findings. For example, a study [17] showed that respiratory problems among radiographers were several times higher than compared group (physiotherapists) with increased respiratory complains within working hours. These increased related symptoms were attributed to the exposures of radiographers to chemical fumes in the darkroom in the mentioned study.

The reported prevalence of the symptom bad chemical taste amongst radiographers was nearly 61.2% of all radiographers, while nurses did not practice such chemical taste at all. Although we didn’t assess the exposure quantitatively in this study, it is well-known that sulphur dioxide, a by-product of the fixation process, is known to be responsible for an unpleasant metallic taste and a bad odour within X-ray departments. The obvious differences between the two professions regarding the prevalence of bad taste should encourage active sulfur dioxide monitoring within X-ray departments. It should be reminded that smoking status was not statistically significant between the two study groups which could minimize or diminish interference and confliction of smoking on results.

### Association of the mean number of symptoms with possible predictors

Many factors were shown to be associated with the mean number of symptoms among radiographers in multivariate linear regression model (see Table 5 for details). These results suggest that safety defensive measures are however not operative in darkrooms in Palestine. Our results could further demonstrate a lack of information about darkroom disease among the Palestinian radiographers. However, a study in Gaza strip found that persons at high risks of developing darkroom disease symptoms were those who spend long periods in diagnostic imaging departments (>10 hours per week) [13]. This conclusion is in accordance with our results that found a significant positive association of the period of stay in darkroom per shift with the mean number of reported symptoms among radiographers. Indeed, our study results were consistent and concurrent with the study conducted by Teschke et al. 2000 [10], which showed that the number of films processed and the time workers spent near the machines increased exposures to chemicals and eventually this was linked to darkroom disease symptoms.

It is of notion that most of radiographers (67.8%) task more than 15 images a day which was found to be positively associated with the mean number of reported darkroom disease symptoms among radiographers. However, in another study carried out in the year 2004, about 8% of the radiographers who reported darkroom disease symptoms were spending an average of 8.8 hours per week in the darkroom [6].

On the other hand, our results revealed no significant differences in the multivariate linear regression model for the association of the mean number of symptoms with the availability of a window in the darkroom among radiographers (95%CI for B, −0.61-3.77). Also, current study findings showed no significant differences in the multivariate linear regression model for the association of the mean number of symptoms with the availability of an extra door in the darkroom among radiographers (95%CI for B, −0.38-2.77). However, the availability of a ventilating machine in the darkroom showed a strong negative association with the mean number of reported symptoms. This coincides with the study conducted by Al Ajerami and Sirdah, 2008 [13], who attributed mainly the exposure of radiographers to chemical fumes in the darkroom to the closed ill-ventilated processing darkrooms and revealed deficiency of quality control measures for darkroom processing in almost 80% of all studied darkrooms, also the authors found a lack of effective departmental ventilation system in almost 73.7% and lack of special dark room ventilation system in almost 78.9% of all studied darkrooms. Hence, different studies concluded that the poor design together with the operational ventilation deficiencies were the major characteristics that resulted in the increased percentages of reported symptoms in such an occupational complex setting [8,13].

In our study, the health complains and problems of radiographers could be attributed to poor ventilation procedures such as weak structural design and deficiencies in operational materials and equipment. Concur to our study results, the study conducted by Taro et al. 2004 [6], showed significant correlation between the darkroom disease symptoms to radiographers and poor design of the radiographic departments presented by mal-ventilation, thereby presenting occupational hazard of chemical exposure to the radiographic personnel. In fact, automatic processors can generate considerable heat to hasten the film development process, thus, it is essential that the darkroom ventilation systems meet the international guidelines.

### Study limitations

This is a recall study where an over or under-estimation of the reported symptoms could have been occurred. Furthermore, our results could have been more consistent if we have performed clinical examinations in parallel to the self-reported symptoms. Furthermore, and although we have used a validated questionnaire, we did not directly ask about the work-relatedness of the participants self-reported symptoms, e.g., “are those symptoms worsened during the working day, working week and/or are they improved during the weekend or during the week absent from work (holidays)”. Therefore, this could be considered as one of the weakness and limitations of this study. Other limitation of this study could be the voluntary participation and the low response rate mainly among nurses which could have led to selection bias on health conditions (either sick or healthy are more willing to participate depending on the context). Clearly enough, a more possible limitation of this study might have also been attributed to the healthy-worker bias where sick workers might have been absent or in vacation so underestimation of the reported symptoms could have been occurred. Due to the study design we have performed, we can’t generate causal relationships between the symptoms of darkroom disease and exposed chemicals in the darkroom. Furthermore, the resulted symptoms could have been attributed to some other factors (living in an industrial area, sharing home with people who smoke) or other confounders that haven’t been studied. One of other shortcomings of this study is that only the radiographers have been asked about the workplace environmental conditions and their effects analysis on symptoms. Therefore, it is possible that the recall bias made symptomatic more prone to answer “yes” also on the worsening factors especially at work.

## Conclusions

Radiographers showed an increase in the prevalence of certain symptoms representing the darkroom disease in comparison with the non-exposed group (hospitals’ nurses). However, trying to interpret this finding in relation to chemicals exposure in their workplace should be done with caution due to the absence of active or passive monitoring in the workplace for the suspected chemicals. The severity of darkroom disease symptoms illustrates the need for legal compliance in order to minimize the occurrence of these symptoms. We recommend educational and training programs for the radiographers and developing clear diagnostic criteria for the darkroom disease. Adoption of digital imaging processes is also necessary.

## Competing interests

The authors declare that they have no competing interests.

## Authors’ contributions

HA drafted the manuscript and participated in the statistical analysis of the data. YN and HA designed the study. YN collected the data and participated in writing the first draft of the manuscript. Both authors read and approved the final version of the manuscript.
